# Ultra-widefield color fundus photography combined with high-speed ultra-widefield swept-source optical coherence tomography angiography for non-invasive detection of lesions in diabetic retinopathy

**DOI:** 10.3389/fpubh.2022.1047608

**Published:** 2022-11-02

**Authors:** Jie Li, Dingyang Wei, Mingzhu Mao, Mengyu Li, Sanmei Liu, Fang Li, Li Chen, Miao Liu, Hongmei Leng, Yiya Wang, Xinru Ning, Yi Liu, Wentao Dong, Jie Zhong

**Affiliations:** ^1^Department of Ophthalmology, Sichuan Provincial People's Hospital, University of Electronic Science and Technology of China, Chengdu, China; ^2^School of Medicine, University of Electronic Science and Technology of China, Chengdu, China; ^3^Eye School, Chengdu University of Traditional Chinese Medicine, Chengdu, China; ^4^Health Management Medical Center, Chengdu First People's Hospital, Chengdu, China; ^5^Department of Ophthalmology, Southwest Medical University, Luzhou, China

**Keywords:** ultra-widefield angiography, color fundus photography, swept-source optical coherence tomography angiography, fundus fluorescein angiography (FFA), diabetic retinopatathy

## Abstract

**Purpose:**

To compare the detection rate of diabetic retinopathy (DR) lesions and the agreement of DR severity grading using the ultra-widefield color fundus photography (UWF CFP) combined with high-speed ultra-widefield swept-source optical coherence tomography angiography (UWF SS-OCTA) or fluorescein angiography (FFA).

**Methods:**

This prospective, observational study recruited diabetic patients who had already taken the FFA examination from November 2021 to June 2022. These patients had either no DR or any stage of DR. All participants were imaged with a 200° UWF CFP and UWF SS-OCTA using a 24 × 20 mm scan model. Images were independently evaluated for the presence or absence of DR lesions including microaneurysms (MAs), intraretinal hemorrhage (IRH), non-perfusion areas (NPAs), intraretinal microvascular abnormalities (IRMAs), venous beading (VB), neovascularization elsewhere (NVE), neovascularization of the optic disc (NVD), and vitreous or preretinal hemorrhage (VH/PRH). Agreement of DR severity grading based on UWF CFP plus UWF SS-OCTA and UWF CFP plus FFA was compared. All statistical analyses were performed using SPSS V.26.0.

**Results:**

One hundred and fifty-three eyes of 86 participants were enrolled in the study. The combination of UWF CFP with UWF SS-OCTA showed a similar detection rate compared with UWF CFP plus FFA for all the characteristic DR lesions (*p*>0.05), except NPAs (*p* = 0.039). Good agreement was shown for the identification of VB (κ = 0.635), and very good agreement for rest of the DR lesions between the two combination methods (κ-value ranged from 0.858 to 0.974). When comparing the grading of DR severity, very good agreement was achieved between UWF CFP plus UWF SS-OCTA and UWF CFP plusr FFA (κ = 0.869).

**Conclusion:**

UWF CFP plus UWF SS-OCTA had a very good agreement in detecting DR lesions and determining the severity of DR compared with UWF CFP plus FFA. This modality has the potential to be used as a fast, reliable, and non-invasive method for DR screening and monitoring in the future.

## Introduction

Diabetic retinopathy (DR) is the most common complication of diabetes and the leading cause of blindness among working-age populations worldwide (1). Screening and prompt treatment of DR play an important role in blindness prevention. The Early Treatment Diabetic Retinopathy Study (ETDRS) established the current standard method for assessing the severity of DR (2, 3). It is based on seven standard retinal fields seen on stereoscopic color fundus photographs (CFPs). The small retinal field covered by conventional ETDRS seven field fundus photographs limits its ongoing clinical application.

In the past decade, retinal imaging technology has evolved rapidly. Ultra-widefield color fundus photography (UWF CFP) taken by scanning laser ophthalmoscope (SLO) could capture fundus images up to 200° and detect DR lesions located in the peripheral retina (4, 5). Previous studies have shown that UWF CFP had a substantial agreement with ETDRS 7-field photographs when determining the severity of DR (5, 6). UWF CFP is becoming increasingly common for rapid screening of diabetic retinopathy. However, the UWF CFP obtained by SLO had some shortcomings, such as pseudo-color, peripheral distortion, image magnification, and lower resolution compared with optical fundus photography (4). Moreover, UWF CFP could not recognize retinal non-perfusion areas (NPAs), and it was difficult to distinguish intraretinal microvascular abnormalities (IRMA) from retinal neovascularization (NV). Therefore, fundus fluorescein angiography (FFA) was a commonly used auxiliary diagnostic method to detect NPAs and other DR lesions that were sometimes difficult to distinguish (7, 8). Thus, UWF CFP combined with FFA was a regular backup option for ophthalmologists or retinal experts in managing DR. Nevertheless, the invasive and time-consuming nature of the procedure and the risk of severe dye-related adverse events limited the use of FFA.

In recent years, swept-source optical coherence tomography angiography (SS-OCTA), as a non-invasive, safe, and easily repeatable retinal blood flow imaging system, was developed. The feasibility of its application in the diagnosis, screening, and follow-up of DR has been evaluated by a large number of studies (9–12). The OCTA equipment used in most of these studies could only capture an area of 12 × 12 mm by a single scan. To expand the range of observation, some studies adopted image mosaic (9, 10), while others used extended field imaging (EFI) technique (13). SS-OCTA image acquired with EFI could capture only slightly wider than the 55° field. On the other hand, Cui et al. used two separate superior and inferior 15 × 9 mm images to achieve a 15 × 15 mm montage image of a 56° field of view (FOV) centered on the fovea (10), and *Khalid et al*. composed five 12 × 12 mm OCTA images to form a montage image of approximately 80° FOV (9). However, the computer built-in Montage scan protocol requires a longer acquisition time and may introduce more artifacts.

The recently developed TowardPi highspeed ultra-widefield SS-OCTA (UWF SS-OCTA) system has an A-scan rate of 400 kHz, and it can obtain a 24 × 20 mm (about 120° FOV) retinal blood flow image within 15 s by a single scan (14, 15). This scanning protocol can display DR lesions, such as microaneurysms (MAs), IRMA, NPAs, and NV. To the best of our knowledge, there is no report on this novel highspeed UWF SS-OCTA system with a 24 × 20 mm scanning area for the detection of DR lesions with a large number of DR cases. Here, we present our preliminary results using this UWF SS-OCTA system to detect DR lesions and to evaluate the idea that UWF CFP plus UWF SS-OCTA could be a fast, reliable, and non-invasive alternative to screen and monitor DR.

## Materials and methods

### Subjects

This prospective, observational study was conducted at the Sichuan Provincial People's Hospital (SPPH) from November 2021 to June 2022. All participants were recruited from patients who have already been scheduled to take the FFA examination (Spectralis HRA + OCT [Heidelberg Engineering, Heidelberg, Germany]) due to various fundus diseases, including DR, macular degeneration, or other retinal disorders. Inclusion criteria: patients with type 1 or type 2 diabetes. These patients may have varying degrees of diabetic retinopathy, including no DR (NDR), non-proliferative diabetic retinopathy (NPDR), and proliferative diabetic retinopathy (PDR). The exclusion criteria were history of glaucoma, concomitant retinochoroidal diseases, the opacity of refractive media affecting more than 30% of OCTA images, and signal intensity of less than 6, poor imaging quality, or serious image artifacts that affect image evaluation. This study was approved by the Institutional Review Board of SPPH and informed consent was obtained from all subjects. All procedures adhered to the tenets of the Declaration of Helsinki and Health Insurance Portability and Accountability Act regulations.

Before the FFA examination, all patients enrolled in the study completed detailed ophthalmic examinations, including best-corrected visual acuity, intraocular pressure, slit lamp anterior segment examination, and slit lamp fundus examination. And UWF SS-OCTA and UWF CPF (California, Optos, Dunfermline, UK) were taken on the same day if they had not taken these two exams within 2 weeks at SPPH.

### Image acquisition protocol

**Figure 1:**
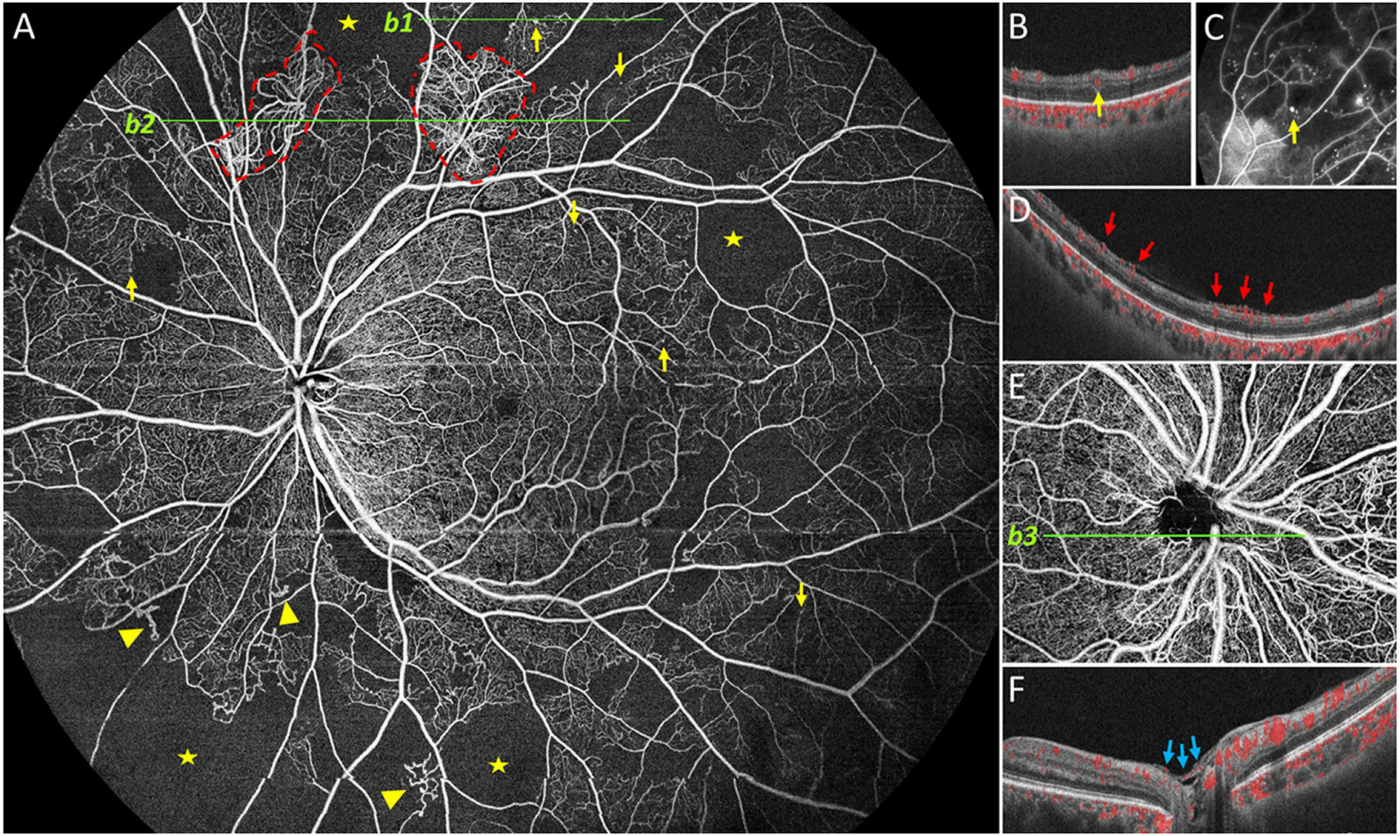
Representative 24 × 20 mm image obtained by UWF SS-OCTA. Representative DR lesions were marked with yellow arrows for microaneurysms (MA), solid triangles for intraretinal microvascular abnormalities, red dashed line for neovascularization elsewhere, pentastar for nonperfusion area, blue arrow for neovascularization of the disc (NVD). In case, neovascularization was found elsewhere on **(A**, *b2*), and corresponding B-scan is provided on **(D)**; B-scan (*b1*) of the MA near the superior edge of **(A)** is shown in **(B)**, and corresponding FFA image is provided in **(C)**. In another case, NVD was found and a corresponding B-scan is shown **(E,F)**.

OCTA images were obtained using a 400 kHz UWF SS-OCTA instrument (BM-400K BMizar, TowardPi Medical Technology, Beijing, China). It uses a swept-source vertical-cavity surface-emitting laser (VCSEL) with a wavelength of 1,060 nm and with a 400,000 A-scans speed, providing a transverse resolution of 10 μm and an axial optical resolution of 3.8 μm. This instrument has an A-scan depth of 6.0 mm in tissue (2,560 pixels). The 24 × 20 mm scan model uses 1,536 A-scans per B-scan at 1,280 B-scan positions, resulting in an A-scan and B-scan separation of 15.625 μm. Two sequential B-scans were performed at each fixed position before proceeding to the next transverse location on the retina. When centered on the fovea, the BM-400K BMizar OCTA imaging is capable of capturing images of retinal blood flow in a range of 24 × 20 mm by a single scan and generate a total FOV of up to 120 degrees (Figure 1A).

The built-in software automatically stratifies the blood vessels in the retina. Each of these segmented volumes is referred to as a slab. Manual correction of retinal segmentation was performed when automatic stratification was inaccurate. The vitreous segmentation and superficial retinal segmentation were selected to detect retinal neovascularization. The superficial retinal slab was defined as the volume between the internal limiting membrane (ILM) and the outer boundary of the inner plexiform layer (IPL). The vitreous slab was defined with an outer boundary positioned on the ILM and with no inner boundary (all the blood flow signals were collected above the ILM).

### Image grading

All images were reviewed independently by two masked ophthalmology specialists. All grading was done on the same monitor, at different time points and in different orders. Disagreements between graders were open adjudicated by an independent senior retina specialist (ZL) who had more than 30 years of working experience in the diagnosis and treatment of DR. UWF CFP and FFA images were analyzed for detecting DR lesions within the 200° FOV and the *en face* UWF SS-OCTA showed about 120° FOV. UWF SS-OCTA images included structural optical coherence tomography (OCT) B-scans with overlay flow signal, and the *en face* OCTA images displayed the superficial capillary plexus and vitreoretinal interface. Based on the three types of images, the two graders identified DR lesions, including MA, intraretinal hemorrhage (IRH), NPAs, IRMA, venous beading (VB), NV of the optic disk (NVD), neovascularization elsewhere (NVE), vitreous or preretinal hemorrhage (VH/PRH), and graded the severity of DR according to the International Classification of DR severity (16), including no DR, mild to moderate NPDR, severe NPDR, and PDR.

All lesions were identified based on their characteristics on OCTA as mentioned in previous reports (9, 10, 17). MAs were defined as moderate or hyperreflective spots with various morphologic patterns, including fusiform, saccular, curved, and rarely a coiled shape (18). MAs arose primarily from the deep part of the inner retinal capillary plexus and were located mostly in the inner nuclear layer. Horizontal OCT B-scans images were used to confirm the presence of Mas, which were identified as capsular shapes and ring signs. Capillary dropouts larger or equal to one-fourth of the disc area were defined as NPAs. IRMAs were tortuous, dilated, and looped intraretinal new vessels adjacent to the areas of capillary loss. VB refers to irregular constriction and dilatation of venules in the retina. NV was observed as extraretinal vessels shown in the vitreoretinal interface slab after segmentation error corrections, and was also defined as a new vessel that has broken through the ILM confirmed by B-scan. NV located in the optic disc or within 1 disc diameter from the margin were classified as NVD, while the rest were classified as NVE. NVD and NVE were confirmed as preretinal hyperreflective material on OCT B-scans.

### Combination of image evaluation and grading

**Figure 2:**
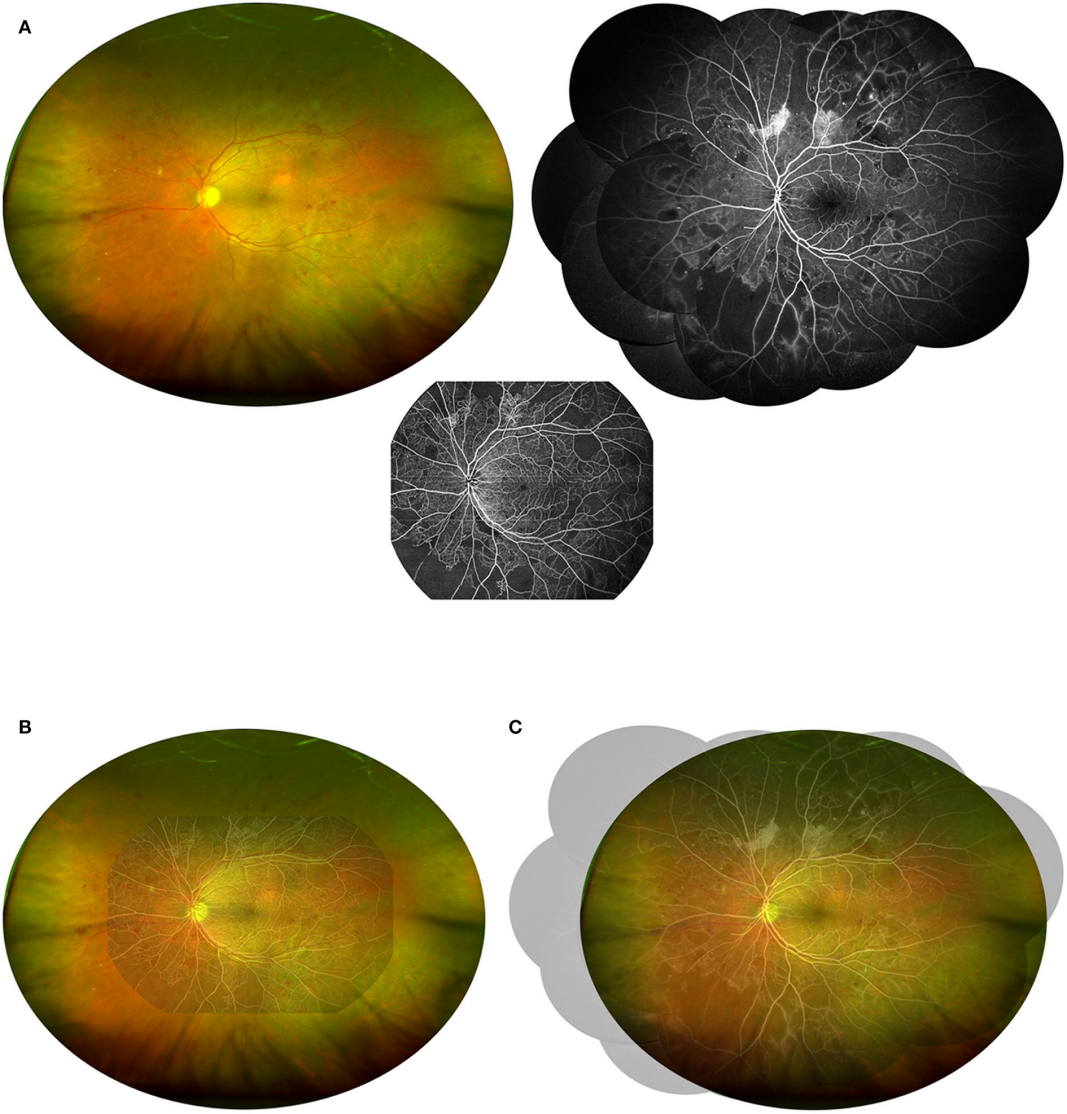
Demonstration of the combination of UWF CFP with UWF SS-OCTA or FFA. **(A)** The UWF CFP, UWF SS-OCTA, and FFA (montaged) images of the same patients shown in Figure 1 were adjusted to a 1:1 ratio and merged in pairs to visually demonstrate the field of view of the two combinations including UWF CFP plus UWF SS-OCTA **(B)** and UWF CFP plus FFA **(C)**.

To compare the detection rate of DR lesions by UWF CFP combined with the other two vascular structure imaging techniques, the evaluation results of UWF CFP were superimposed with the evaluation results of UWF SS-OCTA and FFA (non-invasive versus the other invasive), respectively (Figure 2). The principle of superposition was: if any of the two images of the same eye was positive for a certain DR lesion, the DR lesion of this eye was considered positive. For example, in UWF CFP plus UWF SS-OCTA combination group, if an MA was detected by either of the two methods in one eye, then we considered MA positive for this eye.

### Data analysis

All data were tabulated and organized with Microsoft Excel (Microsoft Corp., Redmond, WA). All statistical analyses were performed using IBM SPSS Statistics 26.0 (IBM Corporation, New York, USA). Normally distributed continuous variables were presented as mean ± SD. DR lesion detection rate differences between different groups were compared using McNemar's test. Inter-rater agreement was calculated using Cohen's Kappa test, wherein a Kappa (κ) value of less than 0.2 means poor agreement, 0.21–0.40 fair agreement, 0.41–0.60 moderate agreement, 0.61–0.80 good agreement, and 0.81–1.00 very good agreement. A nominal two-sided *p-*value of 0.05 was considered to indicate statistical significance.

## Results

### Demographics

A total of 153 eyes (49 eyes with PDR, 56 eyes with severe NPDR, 37 eyes with mild to moderate NPDR, and 11 eyes with diabetic patients with NDR) in 86 participants were enrolled in the study (Table 1). The average age of the participants was 56.7 ± 11.9 years old, and the average duration of diabetes was 10.5 ± 7.6 years. Most of the participants (85/86, 98.8%) had type 2 diabetes mellitus. Except that the macula in three eyes was obscured by vitreous hemorrhage, a total of 98 (65.3%) eyes had diabetic macular edema (DME), of which 67 eyes had center-involved DME.

**Table 1:** Demographics of participants.

Participants (eyes)	153
Mean ± SD age, y	56.7 ± 11.9
Males	53
Females	33
Type of diabetes (patients)	
Type 1	1
Type 2	85
Mean ± SD duration of diabetes, y	10.5 ± 7.6
Groups, severity of DR eyes*	
No DR in DM patients	11
Mild to Moderate NPDR	37
Severe NPDR	56
PDR	49
Right eyes	77
Left eyes	76
Macular edema^#^	
No DME	52
Mild DME or moderate DME	31
severe DME (Center involved DME)	67

*DM*, diabetes mellitus; *DR*, diabetic retinopathy; *NPDR*, non-proliferative diabetic retinopathy; *PDR*, proliferative diabetic retinopathy, *DME*, diabetic macular edema.

* Grading of DR severity based on WF CFP, UWF SS-OCTA, and FFA;

# three eyes with macular was obscured by vitreous hemorrhage.

### Detection rate of DR lesions

#### UWF SS-OCTA vs. FFA

For all DR lesions except NPAs that we investigated, UWF SS-OCTA showed a comparable detection rate with FFA (*p* > 0.05), but less number in MA and VH/PRH and more number in IRMAs, VB, NVD, and NVE. UWF SS-OCTA had better performance in detecting NPAs (73 vs. 69%, *p* = 0.039). Except for VB (κ = 0.279), good or very good agreements were reached for the identification of all DR lesions between UWF SS-OCTA and FFA (κ = 0.791–0.935) (Table 2). There were two eyes with NVE and two eyes with NVD detected by UWF SS-OCTA was defined as NPDR by FFA.

**Table 2:** Detection rate of DR lesions on UWF SS-OCTA vs. FFA.

	**Detection rate of DR lesions on UWF SS-OCTA (24** × **20 mm) vs. FFA (eyes, %)**			
**DR lesions**	**UWF SS-OCTA**	**FFA**	***p*-value**	**κ value**
MA	136/153 (0.89)	140/153 (0.92)	0.125	0.852
IRH	NA	128/153 (0.84)		
NPAs	112/153 (0.73)	105/153 (0.69)	0.039	0.858
IRMAs	99/153 (0.65)	93/153 (0.61)	0.070	0.888
VB	47/153 (0.31)	40/153 (0.26)	0.371	0.279
NVE	41/153 (0.27)	39/153 (0.25)	0.625	0.932
NVD	14/153 (0.09)	12/153 (0.08)	0.500	0.916
VH/PRH	14/153 (0.09)	18/153 (0.12)	0.219	0.791

*DR*, diabetic retinopathy; *MA*: microaneurysms; *IRH*, intraretinal hemorrhage; *NPAs*, non-perfusion areas; *IRMA*, intraretinal microvascular abnormalities; *VB*, venous beading; *NVE*, neovascularization elsewhere; *NVD*, neovascularization of the optic disc; *VH/PRH*, vitreous hemorrhage or preretinal hemorrhage.

#### UWF CFP plus UWF SS-OCTA vs. UWF CFP plus FFA

**Figure 3:**
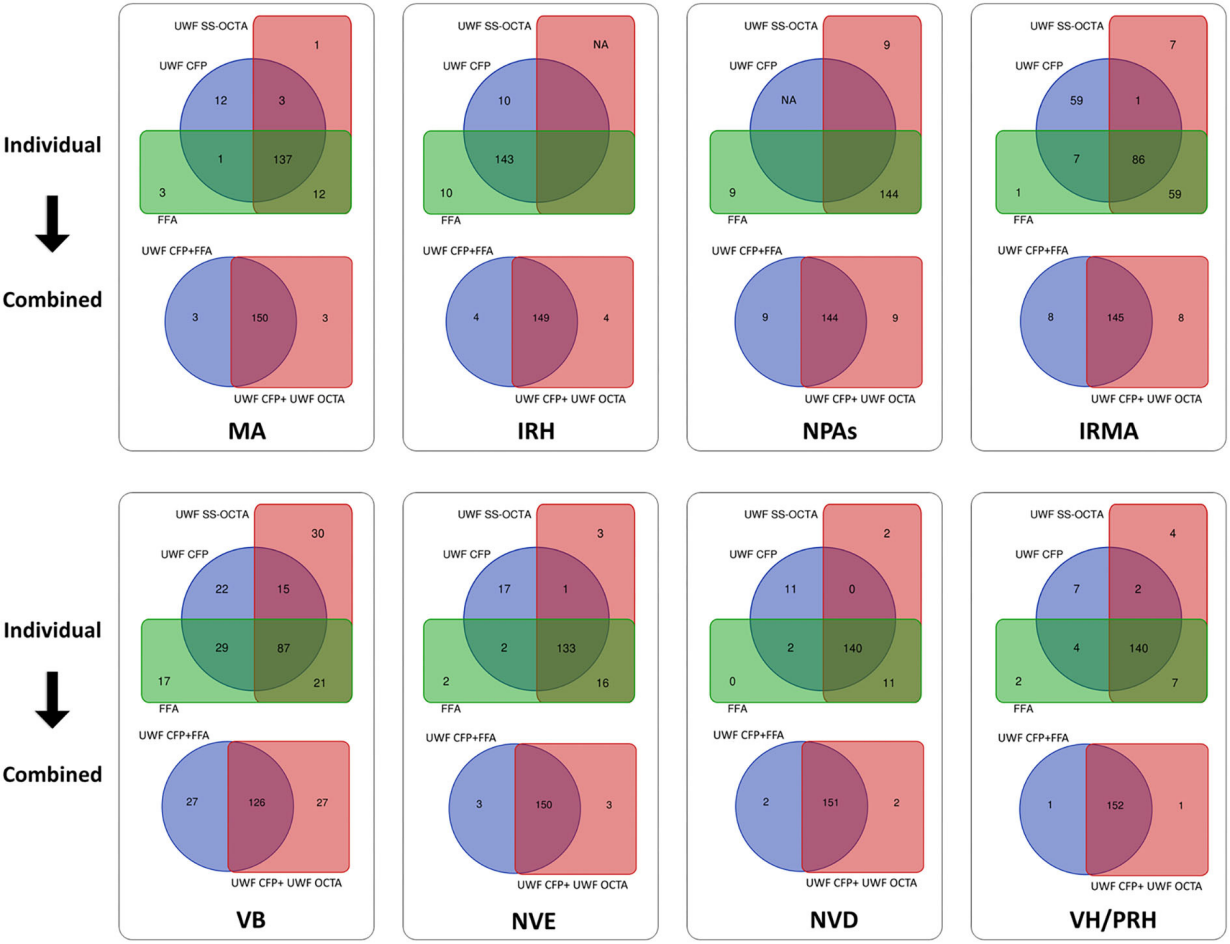
Venn diagram showing the results of two combination methods for detecting DR lesions. The first and third rows show the detection number of each DR lesion by individual modalities, including UWF CFP, UWF SS-OCTA, and FFA. The second and fourth rows show the corresponding combined results of UWF CFP plus UWF SS-OCTA and UWF CFP plus FFA. During the evaluation of the presence and absence of DR lesions, the concordance rates of the three individual modalities from high to low were NP (94.1%), IRH (93.5%), NVD (91.5%), VH/PRH (91.5%), MA (89.5%), NVE (86.9%), VB (56.9%) and IRMAs (56.2%), respectively. And the concordance rates of UWF CFP plus FFA and UWF CFP plus UWF SS-OCTA from high to low were VH/PRH (99.3%), NVD (98.7%), MA (98.0%), NVE (98.0%), IRH (97.4%), IRMAs (94.8%), NPAs (94.1%), VB (82.4%), respectively. MA: microaneurysms, IRH: intraretinal hemorrhage; NPAs: non perfusion areas; IRMA: intraretinal microvascular abnormalities; VB: venous beading; NVE: neovascularization elsewhere; NVD: neovascularization of the disc; VH/PRH: Vitreous hemorrhage or preretinal hemorrhage.

The combination of UWF CFP with UWF SS-OCTA showed a similar detection rate compared with UWF CFP plus FFA for all the characteristic DR lesions (*p*>0.05), except NPAs (*p* = 0.039). Good agreement was shown for the identification of VB (κ = 0.635), and very good agreement for the identification of all the rest of DR lesions (κ >0.8) (Table 3). On the evaluation of the presence and absence of DR lesion, the concordance rates of UWF CFP plus UWF SS-OCT and UWF CFP plus FFA from high to low were VH/PRH (99.3%), NVD (98.7%), MA (98.0%), NVE (98.0%), IRH (97.4%), IRMAs (94.8%), NPAs (94.1%), and VB (82.4%), respectively (Figure 3).

**Table 3:** Detection rate of DR lesions on WF CFP plus UWF SS-OCTA vs. WF CFP plus FFA.

	**Detection rate of DR lesions on WF CFP plus UWF SS-OCTA (24** × **20mm) vs. WF CFP plus FFA (eyes, %)**			
**DR lesions**	**WF CFP plus UWF SS-OCTA**	**WF CFP plus FFA**	***p-*value**	**κ value**
MA	138/153(0.90)	141/153(0.92)	0.250	0.878
IRH	128/153(0.84)	132/153(0.86)	0.125	0.898
NPAs	112/153(0.73)	105/153(0.69)	0.039	0.858
IRMAs	104/153(0.68)	98/153(0.64)	0.070	0.884
VB	67/153(0.44)	56/153(0.37)	0.052	0.635
NVE	42/153(0.27)	41/153(0.27)	1.000	0.950
NVD	14/153(0.09)	12/153(0.08)	0.500	0.916
VH/PRH	22/153(0.14)	23/153(0.15)	1.000	0.974

*DR*, diabetic retinopathy; *MA*, microaneurysms; *IRH*, intraretinal hemorrhage; *NPAs*, non-perfusion areas; *IRMA*, intraretinal microvascular abnormalities; *VB*, venous beading; *NVE*, neovascularization elsewhere; *NVD*, neovascularization of the optic disc; *VH/PRH*, vitreous hemorrhage or preretinal hemorrhage.

### Grading of diabetic retinopathy

#### UWF SS-OCTA vs. FFA

Analysis of UWF SS-OCTA images resulted in a diagnosis of no DR in 15 eyes, mild to moderate NPDR in 35 eyes, severe NPDR in 60 eyes, and PDR in 43 eyes in comparison with 12, 42, 57, and 42 eyes, respectively, when the diagnosis was based on FFA. Of 153 eyes with both UWF SS-OCTA images and FFA images graded, 129 (84.3% [95% CI, 78.6–90.1%]) eyes had an exact agreement. Overall, agreement in grading severity of DR between UWF SS-OCTA and UWF CFP was found to be good (κ = 0.778) (Table 4).

**Table 4:** Agreement in grading DR between UWF SS-OCTA versus FFA.

	**FFA**				
**UWF SS-OCTA**	**No DR**	**Mild to moderate NPDR**	**Severe NPDR**	**PDR**	**Total**
No DR	10	5	0	0	15
Mild to moderate NPDR	2	29	4	0	35
Severe NPDR	0	8	50	2	60
PDR	0	0	3	40	43
Total	12	42	57	42	153

*DR*, diabetic retinopathy; *NPDR*, non-proliferative diabetic retinopathy; *PDR*, proliferative diabetic retinopathy.

#### UWF CFP plus UWF SS-OCTA vs. UWF CFP plus FFA

Analysis of UWF CFP plus UWF SS-OCTA images resulted in a diagnosis of no DR in 13 eyes, mild to moderate NPDR in 31 eyes, severe NPDR in 62 eyes, and PDR in 47 eyes in comparison with 11, 37, 58, and 47 eyes, respectively, when the diagnosis was based on UWF CFP plus FFA. Of 153 eyes graded by both combination methods, 139 (90.9% [95% CI, 86.3–95.4%]) eyes had exact agreement. The overall agreement in grading severity of DR between UWF CFP plus UWF SS-OCTA and UWF CFP plus FFA was found to be very good (κ = 0.869) (Table 5).

**Table 5:** Agreement in grading DR between UWF CFP plus UWF SS-OCTA vs. UWF CFP plus FFA.

	**UWF CFP plus FFA**				
**UWF CFP plus UWF SS-OCTA**	**No DR**	**Mild to moderate NPDR**	**Severe NPDR**	**PDR**	**total**
No DR	11	2	0	0	13
Mild to moderate NPDR	0	30	1	0	31
Severe NPDR	0	5	54	3	62
PDR	0	0	3	44	47
Total	11	37	58	47	153

## Discussion

In this prospective, observational study, we found that UWF SS-OCTA was similar to FFA in detecting DR lesions and grading of DR. At the same time, we found even closer DR lesion detection rate between UW CFP plus UWF SS-OCTA and UW CFP plus FFA. Additionally, a very good agreement was achieved when determining the severity of DR between UW CFP plus UWF SS-OCTA and UW CFP plus FFA. Cui et al. have proposed similar results (10). The difference of this study is that we used a single angiography image of 24 × 12 mm instead of a 15 mm montage image, and we compared the consistency of UWF CFP combined with two angiography methods (one invasive and one non-invasive) in the grading of DR with more cases. Overall, UWF CFP combined with UWF SS-OCTA is a reliable method for the noninvasive and rapid detection of DR lesions.

There have been a large number of previous studies on UWF CFP for the detection of DR lesions. For example, Aiello et al. had revealed that UWF CFP had moderate to a substantial agreement with ETDRS 7-field when determining the severity of DR within the 7 standard fields (6). Moreover, the UWF CFP could detect more lesions outside the 7-field. Silva et al. found approximately one-third of MA, IRMA, and NVE were predominantly outside ETDRS fields and could be detected by UWF CFP (5). The additional peripheral lesions identified by UWF CFP suggested a more severe assessment of DR in 10% of eyes than that of the ETDRS fields. UWF CFP was helpful for the early diagnosis of DR and the detection of more severe lesions. Therefore, UWF CFP has gradually become more and more important in DR screening, diagnosis, and clinical research (19). However, compared with FFA or OCTA, the pseudo-color images of UWF CFP were difficult to distinguish IRMA and NV, and it was almost impossible to complete the task of identifying NPAs. Overall, compared with the two vascular imaging techniques, UWF CFP performed relatively poorly in detecting DR lesions (see Supplementary Tables 1, 2). FFA and recently developed SS-OCTA were therefore needed as a complement, as both are good ways to visualize the subtle vascular structure. In the absence of wide-field OCTA, FFA was the best complementary method to observe DR vascular abnormalities.

FFA is not very prevalent in Western countries in the management of DR, since the impactful ETDRS concluded that color stereoscopic fundus photographs alone were sufficient for DR management (7). Recently, updated guidelines for DR in the United States maintain that routine FFA was not indicated as a part of the regular examination of patients with diabetes (20, 21). However, practice patterns and healthcare delivery systems for patients with diabetes mellitus differ around the world (16). In the past decades, FFA was widely used in China and Japan to diagnose moderate to severe diabetic retinopathy and to help make treatment decisions (8). In our hospital and most other tertiary hospitals in China, outpatient departments were usually crowded with patients, we often routinely screened DR by a rapid UWF CFP and fundoscopy examination. If the patient may have severe NPDR or PDR, FFA would be advised to assist in making the next treatment decision. However, many undeniable shortcomings of FFA including its time consuming and invasive nature, and possible adverse events limited its clinical utility. A non-invasive, rapid alternative to FFA would be ideal for these DR patients. In recent years, with the rapid development of OCTA technology, especially wide-field OCTA, OCTA montage images could observe a much wider range than before. Therefore, a great amount of enthusiasm and resources have been devoted to research in detecting DR lesions using OCTA (17, 22, 23). These previous clinical research results have expanded our understanding of DR, but there were limitations. First, the single scanning range of most previous used OCTA systems was only 12 × 12 mm, which can only cover the posterior 56° of FOV. Second, to get a wider FOV, multiple scanning and montage image were required, which was very time consuming and requires high cooperation from patients (10, 17). Additionally, the EFI technique was introduced to expand the scanning area of OCTA. The mean extension ratio of EFI-OCTA compared to OCTA without EFI was 1.51–1.98, which was reported by different studies (13, 24, 25). However, the EFI-OCTA technique also had several limitations, including a long learning curve, introducing variation in magnification between eyes, and decreasing image resolution.

In this study, we used the latest commercially available high-speed ultra-widefield SS OCTA for the non-invasive detection of DR lesions. The special lens of the TowardPi OCT system is not like the additional lens used in the EFI technique; it is an optical design within a complete set of the system. With a group of large-diameter lenses (ocular lenses more than 60 mm across), the FOV of the system reaches up to 120°, while maintaining a lateral resolution of 10 um (14, 15). The retinal vascular image in the range of 24 × 20 mm can be obtained by a single scan, and the acquisition time is only 15 s at the minimum. It allows technicians to quickly and non-invasively obtain wider and more complete images of retinal angiography image than previous UWF SS-OCTA systems. This undoubtedly improves the efficiency of the examination and makes it easier for patients to cooperate.

**Figure 4:**
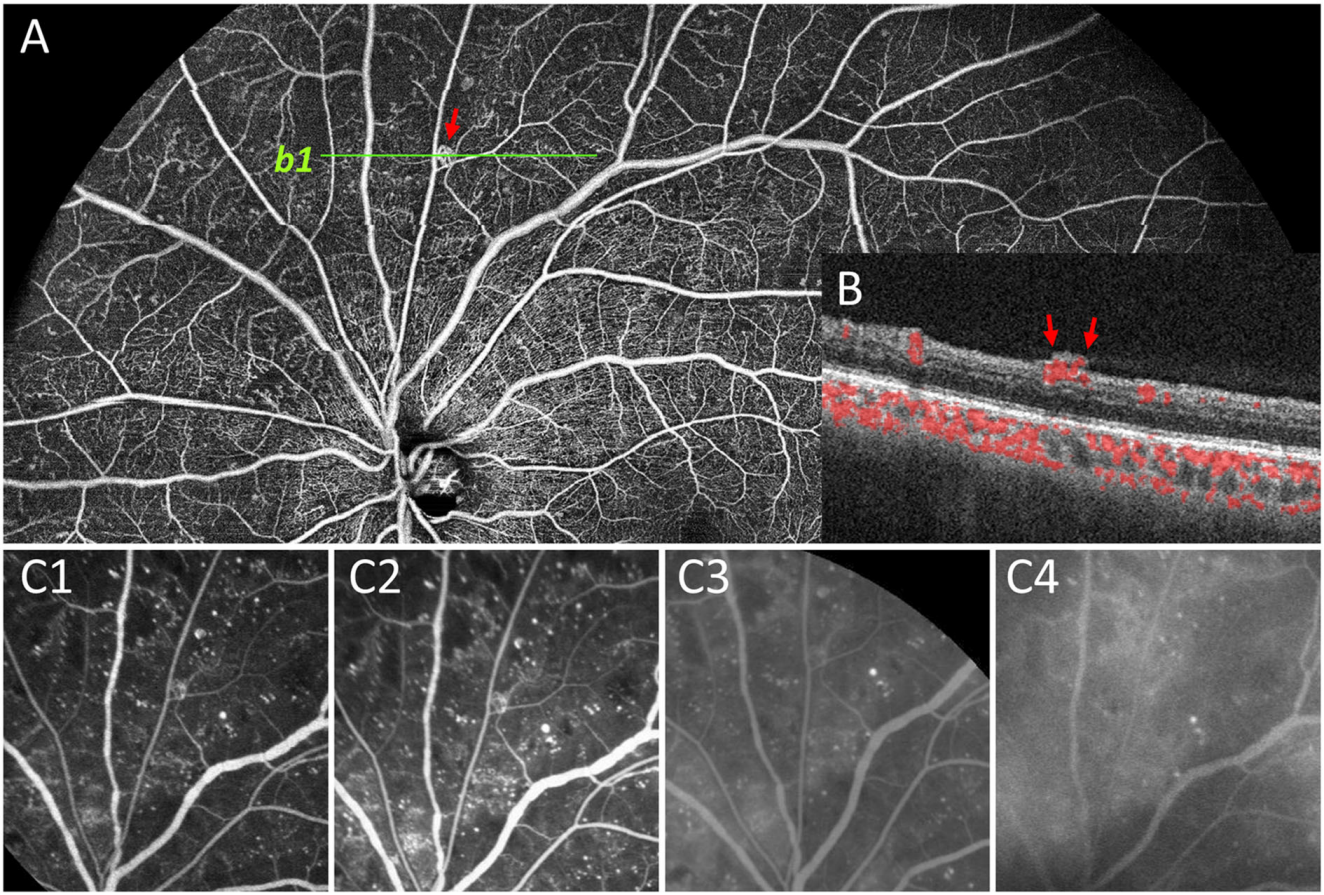
Representative image to show undetected NVE by FFA. In panel **(A)**, the NVE above the optic disc (red arrow) was confirmed by OCT B-scan that the lesion had broken through the ILM (*b1*, **B**). During the whole FFA process **(C1–C4)**, no obvious fluorescein leakage was seen in the corresponding lesion compared with the background leakage. Therefore, the lesion was documented as IRMA rather than NVE on FFA.

**Figure 5:**
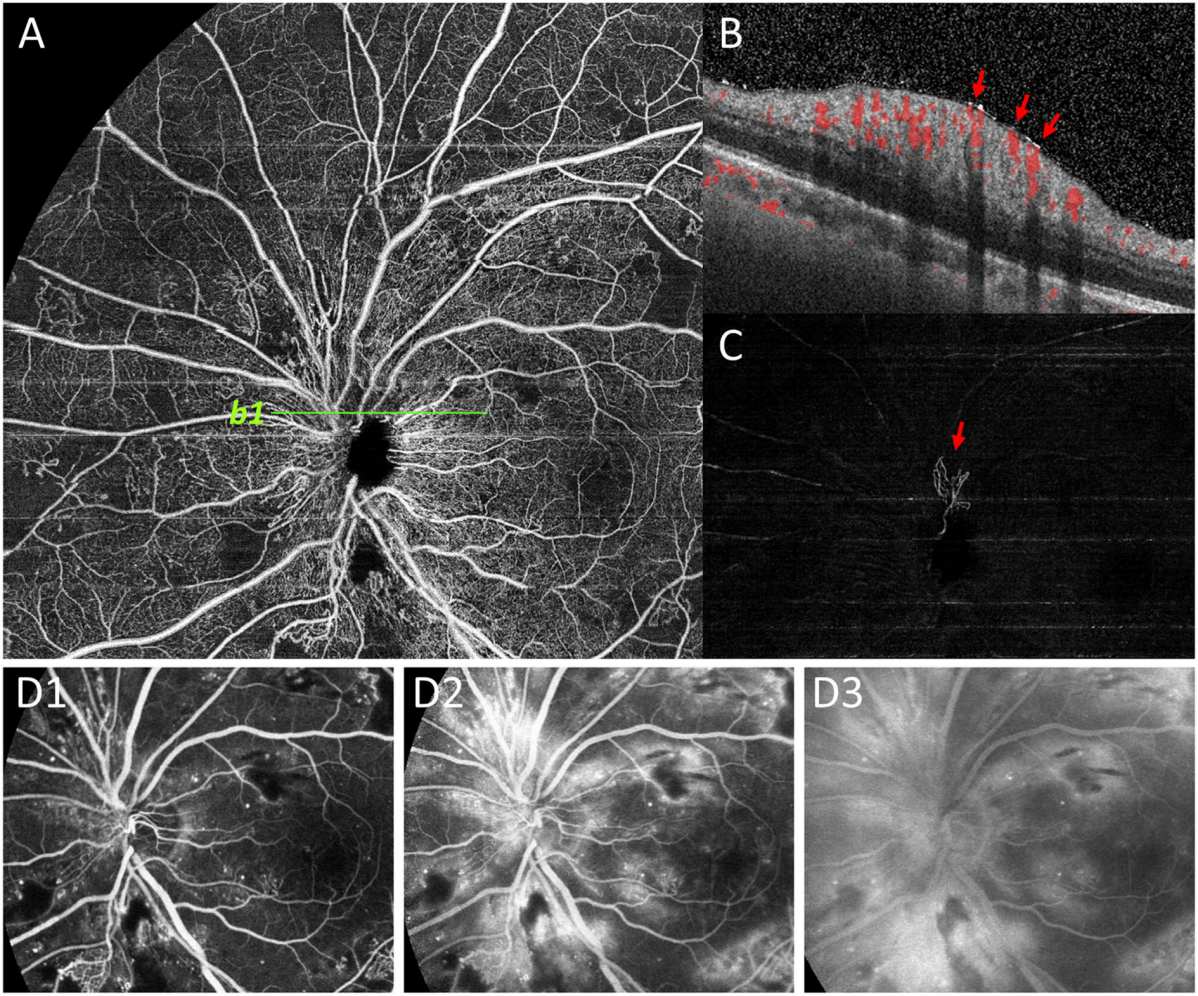
Representative image to show undetected NVD by FFA. NVD was not clearly shown on superficial retinal segmentation of OCTA **(A)**. However, the NVD (red arrow) on the upper edge of the optic disc was clearly shown on the vitreous segmentation **(C)**, and the presence of NVD was also confirmed by B-scan (*b1*, **B)**. On the contrary, due to the large amount of fluorescein leakage around the optic disc area by dilated capillaries on FFA images, the background hyperfluorescence affected the observation of NVD (D1-D3).

For the first time, we used this latest developed UWF SS-OCTA to detect DR lesions in a large number of diabetic patients and compared its practicability with FFA. Our preliminary results showed that there was no significant difference in the detection rate of most DR lesions between UWF SS-OCTA and FFA, and the former had better performance in detecting the rate of NPAs (Table 2). The same result was found when comparing UWF CFP plus UWF SS-OCTA with UWF CFP plus FFA for DR lesion detection (Table 3). During the evaluation of the presence and absence of the DR lesions, the combination methods reached a very high concordance rates in detecting VH/PRH (99.3%), NVD (98.7%), MA (98.0%), NVE (98.0%), IRH (97.4%), IRMAs (94.8%), and NPAs (94.1%) (Figure 3). In addition, there were four eyes with small proliferative lesions (NVE or NVD), which were undetected by FFA and were caught by UWF SS-OCTA (Figures 4, 5). These results indicated that the 24 × 20 mm UWF SS-OCTA was a reliable modality to identify DR lesions, which was similar to previous studies (10, 12). Meanwhile, according to international diabetic retinopathy disease severity scales (DSS), agreement in grading severity of DR between UWF SS-OCTA and UWF CFP was found to be good (κ = 0.778; Table 4). Furthermore, when combined with UWF CFP, UWF CFP plus UWF SS-OCTA and UWF CFP plus FFA had even better performance in DR grading (very good agreement achieved, κ = 0.869; Table 5).

UWF CFP and FFA have been validated by numerous studies in the diagnosis of DR (5–8, 26). Since UWF CFP cannot detect NPAs and the identification of NV and IRMA is often subjective and ambiguous, FFA was used as a complement to color fundus photo to assist DR diagnosis. However, the clinical utility of FFA was limited mainly due to its invasive nature and some other disadvantages. Previous studies and our study showed that compared with FFA, UWF SS-OCTA could also clearly display DR vascular lesions. Moreover, compared with FFA, UWF SS-OCTA had many apparent advantages, such as being fast, non-invasive, repeatable, and inexpensive, which were shared by UWF CFP. Our study confirmed that the grading of DR severity by UWF CFP combined with UWF SS-OCTA was the same as that obtained by UWF CFP plus FFA. Therefore, we suggest that this combination modality might be an optimal choice in the management of DR.

Why do we suggest that UWF SS-OCTA should be used in combination rather than alone for DR screening and follow-up? That's because we have found many shortcomings when we review the UWF SS-OCTA images. For example, the identification of MA and intraretinal hemorrhage on OCTA *en face* images was difficult and sometimes ambiguous. Searching for corresponding lesions on B-scan images was often needed to assist in diagnosis, which was very time consuming. This task would be much easier with the help of UWF CFP images. Furthermore, although 24 × 20 mm is currently one of the widest scanning ranges of commercialized OCTA equipment, many DR lesions are located in the peripheral areas (Figure 2) (27). Therefore, if OCTA, which is more sensitive to NPAs, IRMA, and NV, can be combined with UW CFP, which can more easily identify MA, IRH, and peripheral DR lesions, will undoubtedly improve the reliability of DR diagnosis and grading. Compared with the individual method, the combined methods had a higher concordance rate of DR lesion detection (Figure 3). At the same time, we believe that with the progress of OCTA technology, a combination of both non-invasive imaging techniques has the potential value to gradually replace the majority of FFA examination, and become the first-line tool for fast and large-scale DR screening, diagnosis, and follow-up in the future. A new OCTA-based DR classification consensus would be discussed by international healthcare communities in the near future.

There are limitations to our study. First, the FFA in this study was performed using the 55° field frames instead of an ultra-widefield FFA, which would have lost some clinically significant DR lesions. However, due to the uneven distribution of medical and financial resources, China and many other regions have not yet employed UWF FFA facilities. In addition, the result of our current single-center study needs longitudinal and multi-center studies for further evaluation. Patients with refractive media opacity might have affected the accuracy of OCTA data collection. Compared to FFA, worse contrast sensitivity and motion artifact increased the difficulty to identify MA and VB by OCTA image. In addition, compared to OCTA, we should not ignore the advantages of FFA, which can reveal the destruction of the blood-retinal barrier, and may evaluate iris neovascularization at the same time. Finally, in this Study, we only focused on the evaluation of the presence or absence of DR lesions by the two combination methods, but not studied the number of DR lesions and their implied clinical significance.

In general, UWF CFP combined with UWF SS-OCTA achieved almost the same DR lesion detecting and grading ability as UWF CFP plus FFA. This combination method could be a fast, reliable, and non-invasive method to diagnose DR at all stages, which has been applied in our daily clinical practice and may become a routine choice for DR management in the Future.

## Data availability statement

The original contributions presented in the study are included in the article/Supplementary material, further inquiries can be directed to the corresponding authors.

## Ethics statement

The studies involving human participants were reviewed and approved by institutional review board of Sichuan Provincial People's Hospital (SPPH). The patients/participants provided their written informed consent to participate in this study.

## Author contributions

JZ and JL had full access to the data in the study and take responsibility for the integrity of the data and the accuracy of the data analysis. JZ, JL, and DW concept and designed the research. SL, MLi, DW, LC, WD, MLiu, HL, YW, XN, and YL acquired and sorted the data. MM and MLi reviewed and graded the images. JL, FL, and DW wrote the draft of the manuscript. JZ critically reviewed and extensively revised the manuscript. MM, SL, and MLi performed the statistical analyses. JL, SL, FL, and JZ acquired funding. JZ and WD supervised the study. All authors reviewed the manuscript and provided final approval for submission.

## Funding

This study was supported by the Sichuan Province Central Government Guides Local Science and Technology Development Special Project (2021ZYD0108), the Clinical and Translational Research Fund of Sichuan Provincial People's Hospital (General Project, 2020LY04), the Sichuan Provincial Cadre Health Research Project (ZH2019-201), the Scientific Research Project of Health and Family Commission of Sichuan Province (16PJ454), the Medical science and technology project of Sichuan Provincial Health Commission (21PJ077), and the Chengdu Medical Research Project (2022039).

## Conflict of interest

The authors declare that the research was conducted in the absence of any commercial or financial relationships that could be construed as a potential conflict of interest.

## Publisher's note

All claims expressed in this article are solely those of the authors and do not necessarily represent those of their affiliated organizations, or those of the publisher, the editors and the reviewers. Any product that may be evaluated in this article, or claim that may be made by its manufacturer, is not guaranteed or endorsed by the publisher.
