# Supplementary material for: Ultra-widefield color fundus photography combined with high-speed ultra-widefield swept-source optical coherence tomography angiography for non-invasive detection of lesions in diabetic retinopathy
Source: Front Public Health. 2022 Nov 2;10:1047608. doi: 10.3389/fpubh.2022.1047608 (PMC9667033; doi:10.3389/fpubh.2022.1047608)
Supplement: Supplementary file 1 [file Table_1.DOCX]

**Supplementary material**

Table S1 Detection rate of DR lesion on UWF SS-OCTA versus UWF CFP

UWF SS-OCTA had better performance in detecting IRMA (65 % vs 29 %, p<0.001) and NV (NVE: 27 % vs 17%, p<0.001; NVD: 9 % vs 2 %, p=0.001). On the other hand, UWF CFP was more sensitive than UWF SS-OCTA in detecting VH/PRH (14 % vs 9 %, p=0.039). Additionally, the detecting rate of MA was comparable between UWF SS-OCTA and UWF CFP (89 % vs 84 %, p=0.065). The detection rates of IRH and NPAs were not compared since UWF SS-OCTA was not sensitive to IRH and UWF CFP was not sensitive to NPAs.

| Table S1 Detection rate of DR lesions on UWF SS-OCTA versus UWF CFP | | | | |
| --- | --- | --- | --- | --- |
|  | Detection rate of DR lesions on UWF SS-OCTA versus UWF CFP (eyes, %) | | | |
| DR lesions | UWF SS-OCTA | WF CFP | *p* value | *κ* value |
| MA | 136/153(0.89) | 129/153(0.84) | 0.065 | 0.692 |
| IRH | NA | 128/153(0.84) |  |  |
| NPAs | 112/153(0.73) | NA |  |  |
| IRMAs | 99/153(0.65) | 44/153(0.29) | 0.000 | 0.245 |
| VB | 47/153(0.31) | 37/153(0.24) | 0.203 | 0.184 |
| NVE | 41/153(0.27) | 26/153(0.17) | 0.000 | 0.680 |
| NVD | 14/153(0.09) | 3/153(0.02) | 0.001 | 0.331 |
| VH/PRH | 14/153(0.09) | 21/153(0.14) | 0.039 | 0.711 |
| DR, diabetic retinopathy; MA: microaneurysms; IRH, intraretinal hemorrhage; NPAs, non-perfusion areas; IRMA, intraretinal microvascular abnormalities; VB, venous beading; NVE, neovascularization elsewhere; NVD, neovascularization of the optic disc; VH/PRH, vitreous hemorrhage or preretinal hemorrhage. | | | | |

Table S2 Detection rate of DR lesion on FFA versus UWF CFP

FFA had better performance in detecting MA (92 % vs 84 %, p=0.003), IRMA (61 % vs 29 %, p<0.001) and NV (NVE: 25 % vs 17%, p=0.002; NVD: 8 % vs 2 %, p=0.002). Meanwhile, FFA and UWF CFP had similar detecting rate for VB (26 % vs 24 %, p=0.736) and VH/PRH (14 % vs 12 %, p=0.453), and they had identical detecting rate for IRH (84 % vs 84 %, p=1.000). The detection rates of IRH and NPAs were not compared since UWF CFP was not sensitive to NPAs.

| Table S2 Detection rate of DR lesions on FFA versus UWF CFP | | | | |
| --- | --- | --- | --- | --- |
|  | Detection rate of DR lesions on UWF SS-OCTA (24 mm × 20 mm) versus WF CFP (eyes, %) | | | |
| DR lesions | FFA | WF CFP | *p* value | *κ* value |
| MA | 140/153(0.92) | 129/153(0.84) | 0.003 | 0.605 |
| IRH | 128/153(0.84) | 128/153(0.84) | 1.000 | 0.809 |
| NPAs | 105/153(0.69) | NA |  |  |
| IRMAs | 93/153(0.61) | 44/153(0.29) | 0.000 | 0.293 |
| VB | 40/153(0.26) | 37/153(0.24) | 0.736 | 0.393 |
| NVE | 39/153(0.25) | 26/153(0.17) | 0.002 | 0.671 |
| NVD | 12/153(0.08) | 3/153(0.02) | 0.004 | 0.381 |
| VH/PRH | 18/153(0.12) | 21/153(0.14) | 0.453 | 0.794 |
| DR, diabetic retinopathy; MA: microaneurysms; IRH, intraretinal hemorrhage; NPAs, non-perfusion areas; IRMA, intraretinal microvascular abnormalities; VB, venous beading; NVE, neovascularization elsewhere; NVD, neovascularization of the optic disc; VH/PRH, vitreous hemorrhage or preretinal hemorrhage. | | | | |

Table S3 Agreement in grading DME between UWF SS-OCTA versus FFA

Table S4 Agreement in grading DME between UWF SS-OCTA versus UWF CFP

Table S5 Agreement in grading DME between FFA versus UWF CFP

| Table S3 Agreement in grading DME between UWF SS-OCTA versus FFA | | | | |
| --- | --- | --- | --- | --- |
|  | FFA | | | |
| UWF SS-OCTA | No DME | Mild to moderate DME | Severe NPDR | total |
| No DME | 29 | 20 | 4 | 53 |
| Mild to moderate DME | 1 | 26 | 4 | 31 |
| severe DME | 0 | 4 | 62 | 66 |
| total | 30 | 50 | 70 | 150 |
| DME, diabetic macular edema; severe DME, Center involved DME | | | | |

| Table S4 Agreement in grading DME between UWF SS-OCTA versus UWF CFP | | | | |
| --- | --- | --- | --- | --- |
|  | UWF CFP | | | |
| UWF SS-OCTA | No DME | Mild to moderate DME | Severe NPDR | total |
| No DME | 43 | 10 | 0 | 53 |
| Mild to moderate DME | 18 | 11 | 2 | 31 |
| severe DME | 10 | 41 | 15 | 66 |
| total | 71 | 62 | 17 | 150 |
| DME, diabetic macular edema; severe DME, Center involved DME | | | | |

| Table S5 Agreement in grading DME between FFA versus UWF CFP | | | | |
| --- | --- | --- | --- | --- |
|  | UWF CFP | | | |
| FFA | No DME | Mild to moderate DME | Severe NPDR | total |
| No DME | 30 | 0 | 0 | 30 |
| Mild to moderate DME | 29 | 20 | 1 | 50 |
| severe DME | 12 | 42 | 16 | 70 |
| total | 71 | 62 | 17 | 150 |
| DME, diabetic macular edema; severe DME, Center involved DME | | | | |

The agreement in grading DME between UWF SS-OCTA versus FFA was good (*κ* value: 0.664, Table S3). The agreement in grading DME between UWF SS-OCTA versus UWF CFP was fair (*κ* value: 0.226, Table S4). The agreement in grading DME between FFA versus UWF CFP was fair (*κ* value: 0.216, Table S5)
